# Predictors of FIFA 11+ Implementation Intention in Female Adolescent Soccer: An Application of the Health Action Process Approach (HAPA) Model

**DOI:** 10.3390/ijerph13070657

**Published:** 2016-07-07

**Authors:** Carly D. McKay, Charlotte K. Merrett, Carolyn A. Emery

**Affiliations:** 1 Department for Health, University of Bath, Bath BA2 7AY, UK; 2 Sport Injury Prevention Research Centre, University of Calgary, Calgary, AB T2N 1N4, Canada; caemery@ucalgary.ca; 3 Department for Health, University of Bath, Bath BA2 7AY, UK; c.k.merrett@bath.ac.uk; 4 Faculty of Kinesiology, University of Calgary, Calgary, AB T2N 1N4, Canada

**Keywords:** adolescent, injury prevention, soccer

## Abstract

The Fédération Internationale de Football (FIFA) 11+ warm-up program is efficacious at preventing lower limb injury in youth soccer; however, there has been poor adoption of the program in the community. The purpose of this study was to determine the utility of the Health Action Process Approach (HAPA) behavior change model in predicting intention to use the FIFA 11+ in a sample of 12 youth soccer teams (coaches *n* = 10; 12–16 year old female players *n* = 200). A bespoke cross-sectional questionnaire measured pre-season risk perceptions, outcome expectancies, task self-efficacy, facilitators, barriers, and FIFA 11+ implementation intention. Most coaches (90.0%) and players (80.0%) expected the program to reduce injury risk but reported limited intention to use it. Player data demonstrated an acceptable fit to the hypothesized model (standardized root mean square residual (SRMR) = 0.08; root mean square of error of approximation (RMSEA) = 0.06 (0.047–0.080); comparative fit index (CFI) = 0.93; Tucker Lewis index (TLI) = 0.91) Task self-efficacy (β = 0.53, *p* ≤ 0.01) and outcome expectancies (β = 0.13 *p* ≤ 0.05) were positively associated with intention, but risk perceptions were not (β = −0.02). The findings suggest that the HAPA model is appropriate for use in this context, and highlight the need to target task self-efficacy and outcome expectancies in FIFA 11+ implementation strategies.

## 1. Introduction

The Fédération Internationale de Football (FIFA) estimates that there are over 22 million youth soccer players globally [1], and the frequency of injury in this population represents a significant public health burden. Estimated injury rates in youth soccer range from 3.4–5.6 injuries/1000 participation hours, or 22.0–30.0 injuries/100 participants/year [2,3,4]. These are characterized by a high proportion of lower extremity injuries, with the majority being to the ankle and knee joints [2,3,4,5,6,7,8,9]. Aside from their immediate impact on individual health, there is evidence that joint injuries result in an increased risk of early osteoarthritis development [10,11,12] and may have lasting impacts on physical activity participation and health-related quality of life [12]. Implementing successful injury prevention strategies in this population is, therefore, critical in reducing health care costs and decreasing risks for disability and inactivity through adulthood. 

Exercise-based prevention programs are efficacious at reducing sport injury risk [13,14,15,16]. Neuromuscular training programs, in particular, have demonstrated significant lower extremity injury reductions in youth sport (incidence rate ratio (IRR) = 0.6; 95% confidence interval (CI): 0.5, 0.8) [15,17]. The FIFA 11+ is a neuromuscular training program designed specifically to reduce lower extremity injuries in amateur soccer, and in randomized trials has produced significant risk reductions of 32.0%–56.0% [18,19,20]. Previous studies, however, have reported only moderate adherence to the FIFA 11+ and, despite its efficacy, there has been limited uptake of the program in the soccer community [21]. This reflects many degrees of influence for program implementation including the broader sport culture as well as institutional/organizational, team, and individual levels. Although the most effective driver for implementation may come from institutional change [21], this process often takes significant time and resources and there remains the challenge of modifying individual behavior in the absence of enforced program use. Effecting change at this level relies on an understanding of the determinants of program uptake in the target population, including barriers and facilitators to its use in context. 

A number of barriers to FIFA 11+ use have been identified amongst youth soccer players and their coaches, including low expectation that prevention strategies are effective and having insufficient time or resources to implement the program [22]. Notably, promotion strategies for prevention programs often rely on communicating injury risk information to the target audience. In youth soccer, however, knowledge of injury rates and perceptions about whether injuries are preventable have demonstrated poor associations with program uptake [23]. Furthermore, factors such as personal injury history, which should provide realistic risk perceptions, have not been supported as adherence predictors [23]. Such counterintuitive findings may be related to the absence of an underlying behavioral theory in the majority of existing research. Cataloguing perceptions and potential barriers without establishing how they interact with cognitive processes to produce behavioral intentions and actions limits our understanding of program adoption in the soccer community.

Prevention program delivery must also be considered when evaluating adherence. In both research and practical settings, emphasis has been placed on providing injury prevention content to coaches who can then disseminate information to players. This has been moderately successful [18,19], but results of a recent study investigating different FIFA 11+ delivery strategies suggest that program adherence increases by 8.0%–12.0% when coaches are given comprehensive, workshop-style instruction instead of simply providing program resources [20]. Understanding the underlying determinants of behavior change may help to explain this finding and better inform the content of coach workshops to promote greater program uptake and maintenance in the face of identified barriers to program use.

Behavior change theory has seen limited application in sport injury research [24,25]. In a systematic review, McGlashan and Finch report that only 11.0% of prevention studies included explicit application of a behavioral or social science theory, and only four of 11 total papers formally tested a theory-driven hypothesis [24]. A number of common behavior change theories were notably absent from the literature. Given the limited success in promoting prevention program uptake in sport settings, there is considerable scope for additional work in this area to facilitate behavior change at individual and team levels [24].

The Health Action Process Approach (HAPA) model is a theory of health behavior change that is particularly suited to describing the adoption of preventive behaviors [26]. It comprises a motivation phase during which individuals form a behavioral intention, and a two-stage goal-pursuit (volitional) phase where intenders form plans to enact, and actors actually engage in, a target behavior. In the motivation phase, perceived task self-efficacy and outcome expectancies are posited as major predictors of intention, with risk perception exerting a minor direct influence [26]. In the volitional phase, intention must be translated into action through a process of action and coping planning, influenced by perceived maintenance self-efficacy. Once the behavior has been initiated, perceived maintenance and recovery self-efficacy govern self-regulatory processes that can be influenced by environmental barriers and facilitators that can promote or inhibit continued action. The HAPA model has demonstrated utility in predicting and directing behavior in a number of health domains, including health screening, diet, and sunscreen use [27,28,29], and has been advocated as a framework for designing health promotion interventions. 

Although other theories of behavior change have been explored in injury prevention [24,25], many focus on social cognitive processes that originate well before the behavior in question (e.g., motivation) and are, therefore, unable to account for substantial amounts of variance when modeled in real world settings. The HAPA model, however, is centered on more proximal constructs that can be readily measured and may be amenable to change in a short period of time [26]. Additionally, whereas other models (e.g., Theory of Planned Behavior) are oriented toward volitional processes for undertaking positively- framed behaviors, such as exercise for health benefit or enjoyment, injury prevention is inherently an avoidance-framed action and, therefore, suited to models that account for individual perceptions of risk and outcome expectancy. The HAPA model also satisfies this criterion. Moreover, the model describes the influence of factors that influence behavioral maintenance following initial uptake, which is appropriate considering the dose-response nature of the FIFA 11+ [19]. This added predictive power represents an incremental step beyond previously explored models, and as such could provide additional insight into the implementation challenges faced in sport injury prevention contexts. 

Therefore, the purpose of this study was to explore the utility of the HAPA model in predicting intention to adopt the FIFA 11+ amongst youth soccer coaches and players, as an initial proof of concept to inform the design of larger prospective studies. The primary objective was to describe perceived task self-efficacy, outcome expectancies, and risk perceptions in this setting. The secondary objectives were to determine whether these factors predicted intention to use the FIFA 11+ at the start of the soccer season and to assess the fit of the HAPA model as an appropriate behavior change theory to apply in this context. 

## 2. Materials and Methods 

### 2.1. Study Design

This was a cross-sectional study conducted at the start of the 2013 outdoor soccer season (May–August) in Calgary, Canada. This study was approved by the Conjoint Health Research Ethics Board at the University of Calgary (No. 24985).

### 2.2. Participants

The study population consisted of coaches and youth female soccer players (aged 12–16 years) competing in the Calgary Minor Soccer Association. Due to the exploratory nature of the study, no formal *a priori* sample size was calculated. Coaches were eligible to participate if they were the head or assistant coach of a female soccer team competing in Tier 1 or 2 of the U14 or U16 age group at the start of the season, and were excluded if they had any previous experience using the FIFA 11+. Players were eligible if they belonged to a female soccer team competing in the U14 or U16 age group at the start of the season and their team coach was participating in the study (as coach permission was required to approach players for recruitment). Exclusion criteria for players included a recent (within six weeks) history of injury requiring medical attention or the inability to participate in soccer for at least one day and preventing full participation at the start of the 2013 season, or a history of systemic disease or neurological disorder preventing full participation at the start of the 2013 season. Teams were randomly approached for recruitment by club once teams were formed prior to the start of the season. All coaches provided written informed consent, and players provided written informed parental consent and personal assent to take part in the study.

### 2.3. The FIFA 11+ Program

The FIFA 11+ program is a 20-min warm-up that consists of running exercises, dynamic stretching, plyometrics, and agility, strengthening, and balance exercises [30]. It was developed specifically to reduce the risk of lower limb injury amongst recreational soccer players, and as such, the focus of the program is on cutting, jumping, and landing technique. The program is freely available online and is supported by a suite of resources, including a coaching manual, videos, posters, and flashcards [30]. 

### 2.4. Procedure

Prior to the start of the season, coaches attended a two-hour FIFA 11+ instructional workshop, delivered by the lead author (who has experience playing and coaching soccer at the level targeted in this study). Each exercise in the program was demonstrated, with clear directions on identifying correct and incorrect technique, and coaches were given copies of the program resources. The emphasis of the workshop was on the program content, with only a brief rationale for its use (e.g., a why the program was developed and a short summary of evidence for its efficacy) presented for context. Coaches were clearly told that choosing to use the FIFA 11+ in the following season would be entirely voluntary, and the purpose of the study was to better understand why teams might or might not decide to use the program. 

After this familiarization, coaches completed a questionnaire to assess demographic characteristics, perceptions about soccer injury, and the HAPA constructs. 

Player questionnaires were administered by study personnel in a supervised team setting at a training session prior to the start of the season. Coaches explained the FIFA 11+ to the players, and the questionnaires were answered based on the players’ understanding of the program at that time.

### 2.5. Outcome Measures

A bespoke questionnaire was developed for this study, with separate versions for coaches and players (see supplementary online content). Section A of the questionnaire captured demographic, soccer coaching/playing experience, and injury history information. Section B focused on the HAPA constructs, with questions assessing self-efficacy, outcome expectancies, risk perceptions, behavioral intentions, and barriers and facilitators to FIFA 11+ implementation. 

Questions were developed by adapting previously validated HAPA questionnaire item stems [31] to reflect the youth soccer context. For example, the stem “How serious are the following health-related problems?” with a response list including heart attack, stroke, and high blood pressure [31] was adapted to “How serious are the following types of soccer injury?” with a response list including ankle sprain, knee ligament injury, and broken bone (with both questions scored on a seven-point Likert scale ranging from *not at all serious* to *very serious*) (see supplementary content). The questionnaires underwent face validation by a group of experts (a soccer coach, a youth soccer player, a sport psychology researcher, and a physiotherapist) who were all familiar with the FIFA 11+ program. Questions were answered on a seven-point Likert scales that included reverse-coded items to ensure accurate completion. For analysis purposes, items were scored on a scale of 1 = ‘extremely negative’ to 7 = ‘extremely positive’.

### 2.6. Analysis

Descriptive analyses (frequencies, means, standard deviations (SD)) were used to assess demographic characteristics and questionnaire responses. Due to the low number of coaches in the sample, data were checked for normal distribution and Pearson correlations were subsequently used to assess the association between HAPA variables and intent to use the FIFA 11+. The threshold for statistical significance was set at *p* = 0.05 and, due to the exploratory nature of the study, no corrections were made for multiple comparisons. These analyses were conducted using STATA v.13 (StataCorp; College Station, TX, USA).

To assess the fit of the HAPA model for predicting player intention, structural equation modeling was employed [32] and a two-step approach was applied [33]. The standardized root mean square residual (SRMR) and the root mean square of error of approximation (RMSEA) were chosen as indicators of absolute fit [34]. SRMR and RMSEA values ≤0.08 and ≤0.06, respectively, have been deemed to indicate a model with good fit to the data [34]. The incremental fit indices were represented by the comparative fit index (CFI) and the Tucker Lewis index (TLI). Values >0.90 indicate adequate model fit, and values >0.95 are considered to indicate excellent fit [34]. Modeling was conducted in AMOS v.22 (SPSS; Chicago, IL, USA).

## 3. Results

Of 40 teams approached for recruitment, 12 consented to participate in the study. One team was excluded because the coach had experience with the FIFA 11+, 22 responded that they were not interested in engaging in research, and five were unable to schedule a data collection session prior to the start of the season and so declined participation. Two consenting coaches did not attend the pre-season workshop, leaving a final sample of 10 coaches. Of the 203 eligible players, all were successfully recruited but only 200 provided complete questionnaire responses and were retained for analysis. Participant characteristics are presented in Table 1. Six of the coaches, and just over half of the players (58.1%), reported using injury prevention strategies during the previous season, comprised of general fitness training and regular team warm up activities (e.g., jogging, ball games). Of the reported injuries in the previous year, 15 (20.0%) were ankle injuries and 22 (29.3%) were knee injuries.

### 3.1. Perceived Injury Risk and Expectancies

**Figure 1:**
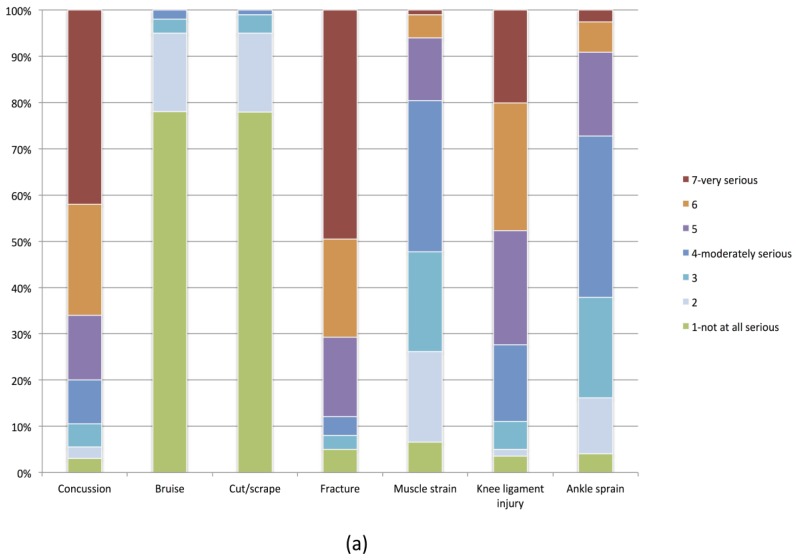

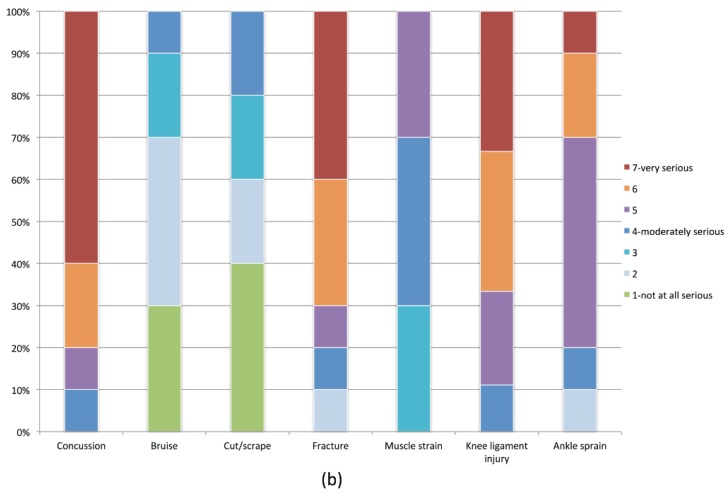
(**a**) Player perceptions of the severity of different injury types (1 = not at all serious, 7 = very serious) (% reporting each category); (**b**) Coach perceptions of the severity of different injury types (1 = not at all serious, 7 = very serious) (% reporting each category).

Baseline injury risk perceptions and prevention expectancies are presented in Table 2. Most coaches (80.0%) believed injuries were likely to occur in youth soccer, and 44.5% of players expected to sustain an injury during the following season. A greater proportion of coaches (60.0%) and players (47.5%) thought that knee injuries were “quite” or “very” serious when compared to ankle injuries (coaches: 30.0%, players: 9.0%), but both groups rated concussions and fractures as the most serious injury types (Figure 1). 

Overall, 44.0% of players believed injuries were ‘quite’ or ‘definitely’ preventable, whereas coaches generally believed they were only ‘slightly’ preventable. The majority of coaches (90.0%) and players (80.0%) expected that the FIFA 11+ would reduce injury risk if used at every training and game session. 

### 3.2. Perceived Self-efficacy

Perceived self-efficacy is presented in Table 3. Both groups reported only slight confidence in their abilities to use the FIFA 11+ for the entire upcoming season, and demonstrated limited confidence for recovering following a missed session.

### 3.3. Facilitators and Barriers to FIFA 11+ Use

The most commonly-reported facilitators for program use amongst coaches were access to FIFA 11+ materials (e.g., videos, posters) (endorsed by 100% of coaches), having the proper equipment (40.0%), and having enough space to accommodate the exercises (20.0%). The most common barriers were not having enough time (40.0%), poor player buy-in/lack of player cooperation (40.0%), and coaching staff absences (20.0%). For players, the most common facilitators were personal motivation/willingness to put in effort (35.5%), having enough time (31.5%), making the program a routine (29.0%), and someone taking responsibility for organizing and leading the warm-up (29.0%). Player barriers included poor team buy-in to the program (52.0%), finding the program too difficult/tiring (35.5%), and low personal motivation (29.0%). A complete list of barriers and motivators can be found in the Appendix (Table A1 and Table A2).

### 3.4. Intention to Use the FIFA 11+

Coaches reported limited intention to make injury prevention a priority in the upcoming season (mean = 5, SD = 1.5; range 1–6) or to use the FIFA 11+ with their team (mean = 5, SD = 1.2; range 2–6). Similarly, players reported no distinct intention to use the FIFA 11+ at every session during the season (mean = 4.6, SD = 1.2; range 1–6), or to perform the exercises with full effort if they did use the program (mean = 4.7, SD = 1.2; range 1–6). 

The associations between HAPA predictor variables and intention for coaches are presented in Table 4. Self-efficacy related to understanding the program (*r* = 0.84, *p* ≤ 0.01) and self-efficacy for using the program (*r* = 0.89, *p* ≤ 0.01) were the only variables significantly associated with intention.

**Figure 2:**
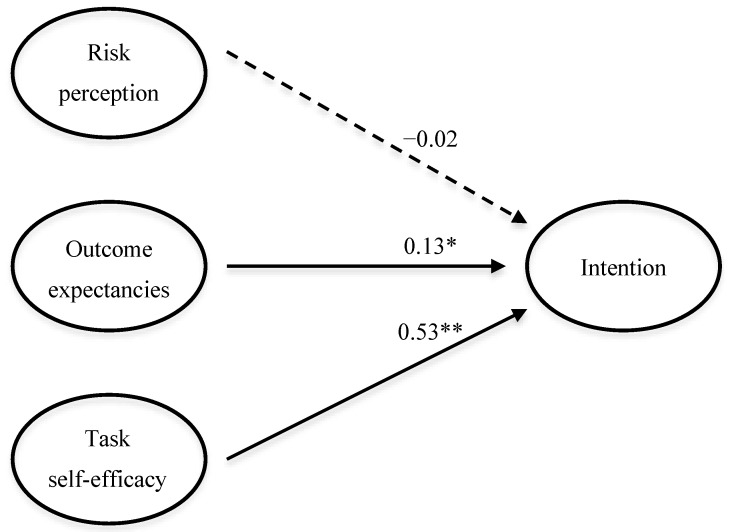
Simplified structural equation model demonstrating HAPA prediction of player intention to adopt the FIFA 11+ program in the following season (* significant at *p* ≤ 0.05; ** significant at *p* ≤ 0.01). All path coefficients are standardized.

Player data demonstrated good fit to the hypothesized HAPA prediction model (SRMR = 0.08; RMSEA = 0.06 (0.047–0.080); CFI = 0.93; TLI = 0.91) (Figure 2). Overall, player task self-efficacy was positively associated with intention to adopt the FIFA 11+ (β = 0.53, *p* ≤ 0.01). Outcome expectancies had a small but positive significant effect on intention (β = 0.13 *p* ≤ 0.05), but risk perceptions demonstrated no association (β = −0.02). 

## 4. Discussion

### 4.1. Injury Risk, Outcome Expectancies, and Self-Efficacy

Consistent with previous studies in this population [22,23], coaches had higher general risk perceptions than players. Both groups rated knee ligament injuries as more serious than ankle sprains, but thought that fractures were more severe than either. Although coaches and players are typically aware of lower limb injury risk [22,23], they may not view ankle injuries, in particular, as particularly severe. Prevention messaging focusing on reducing these injuries may, therefore, be less effective than intended. 

A larger proportion of coaches and players believed that injuries might be preventable in the present study than in previous studies in the same population [22]. Furthermore, 80.0%–90.0% of the present sample believed that the FIFA 11+ would effectively prevent injuries if used throughout the season. This may reflect increasing awareness of the FIFA 11+ amongst stakeholders resulting from ongoing promotion activities in soccer clubs [21]. 

Self-efficacy for using the program, however, was low amongst coaches and players. Bandura [35] posits that the main sources of self-efficacy are mastery experience, vicarious experience, verbal persuasion, and internal physiological states. Given that the present sample had not used the FIFA 11+ prior to the study, the only major sources of confidence would be knowledge of similar teams finding the program effective or from verbal assurances during the workshop sessions. Self-efficacy is a strong determinant of behavior in exercise and other health domains [36,37], and has been shown to be the driving behavioral predictor in the HAPA model [26,27,28,29]. Providing self-efficacy-enhancing activities in instructional workshops, such as hands-on practice teaching (coaches) or performing (players) the exercises is, therefore, a useful avenue to explore for increasing intention to use the program. Including testimonials from other teams, or using mentorship schemes within clubs, may also be successful strategies for improving program uptake.

### 4.2. Facilitators and Barriers 

Program length, lack of time and space, and limited exercise variety have been reported as barriers to prevention program uptake in several sports [38,39]. Participants in the present study identified many of the same concerns, suggesting that injury prevention initiatives across contexts face the same challenges. Notably, facilitators for FIFA 11+ use for coaches (access to FIFA 11+ instructional resources, equipment to set up the program, enough space) were largely practical in nature, as were identified barriers (not enough time, coaches absent). Players, however, identified more interpersonal facilitators (personal motivation, development of routines, leadership) and barriers (low personal interest, poor team cooperation, perceived physical difficulty of the exercises). These differences between coach and player perceptions indicate that barrier mitigation strategies based on coach recommendations are likely to miss significant team-level issues (such as player support of the program). Considering that players indicated low intention to perform exercises with full effort even if their team did use the FIFA 11+, and coaches identified poor player buy-in as a significant barrier, addressing interpersonal factors in coach workshops may improve overall program adoption and enhance the quality of exercise performance. 

### 4.3. Intent and the HAPA Model

Although the coach sample was too small to conduct a factor analysis, item-wise correlations suggest that only task self-efficacy was significantly associated with intention. For players, outcome expectancies and task self-efficacy were related to intention, while risk perceptions were not. The relative strengths of these relationships are consistent with the HAPA literature, with self-efficacy being the strongest predictor of intention and risk perception contributing only a small amount to the overall model [26,27,28,29]. It also supports previous findings that self-efficacy is related to safety behaviors in other sports [40]. For coaches there was a notable, but not statistically significant, association between perceived knee injury severity and intention (*r* = 0.55). This could point to a meaningful contribution of specific risk perceptions to FIFA 11+ adoption, but should be interpreted with caution given the small sample size. Moreover, although no direct relationship emerged between risk perceptions and intention in the full player model, previous studies suggest that low risk perceptions are related to limited intention to engage in injury prevention behaviors [41,42,43]. The relatively low risk perceptions in this sample may, therefore, have prevented the detection of significant relationships between these variables. 

Based on the fit indices of the player model, it appears that HAPA is a suitable framework in this context. Additional research will be needed to better quantify the strength of the relationships between predictor constructs and intention, and the volitional stages of the model must be investigated to determine whether it can be used to predict behavior. Yet, the results of the present study suggest that FIFA 11+ promotion strategies that focus on injury risk education may be largely ineffective [44]. This may help to explain why coach workshops that include practical instruction are more effective than simply providing coaches with resource materials [20]. Including players or player representatives in these workshops could enhance this effect even further by fostering a program-supportive team climate and encouraging greater coach and athlete self-efficacy for teaching and performing the exercises.

### 4.4. Limitations and Future Directions

A number of limitations to this study must be acknowledged. First, the questionnaires have not undergone a rigorous validation process. Although they performed reasonably well for exploratory purposes, it is possible that the associations under investigation were under- or over-estimated due to imprecision in the measurement. Furthermore, the small sample of coaches prevented analysis of the model fit for this group. Future studies should, therefore, refine the questionnaires and seek to validate them in larger samples. This study also used intention as an end point, and there is a widely acknowledged gap between intention and behavior in prevention program implementation [45]. While the results have provided initial proof of concept that the HAPA model is appropriate in this setting, future research must, therefore, investigate its utility in predicting actual FIFA 11+ adoption by measuring program use during the season.

Finally, in youth team sports, coach intentions likely influence team behaviors more than those of individual athletes. Therefore, better understanding how coach perceptions match with the perceptions of their players, or how information is communicated between coaches and players, may help to inform FIFA 11+ delivery strategies and provide greater insight into the appropriate content and audience for instructional workshops. Developing workshop content based on the HAPA constructs (e.g., that promotes realistic outcome expectancies and strengthens self-efficacy) will also be important moving forward.

## 5. Conclusions 

This is the first study to examine the HAPA model in predicting injury prevention intention in female youth soccer. The findings suggest that the model is indeed appropriate for use in this context, and highlights the strong relationship between task self-efficacy and intention to use the FIFA 11+ warm up program. This study has also demonstrated that perceived facilitators and barriers to program adoption differ between coaches and players, providing possible avenues for improving program delivery in the community.

## Figures and Tables

**Table 1:** Participant characteristics.

Characteristic	Coaches (*n* = 10)	Players (*n* = 203)
	Frequency (%)	Frequency (%)
**Age group**		
***U14***	6 (60.0)	136 (67.0)
***U16***	4 (40.0)	67 (33.0)
**Competitive tier**		
***I***	5 (50.0)	115 (56.7)
***II***	5 (50.0)	88 (43.3)
**Used injury prevention strategies last season ^1^**	6 (60.0)	118 (58.1)
**Injury in the previous year**	-	75 (36.9)
***Lower limb***	-	62 (82.7)
***Other***	-	13 (17.3)

^1^ General fitness training and team warm-ups consisting of jogging and ball games; no participant reported using neuromuscular training/FIFA 11+ exercises.

**Table 2:** Baseline injury prevention expectancies.

Expectancies	Frequency (%)						
	1	2	3	4	5	6	7
	Very Unlikely	Quite Unlikely	Slightly Unlikely	Neutral	Slightly Likely	Quite Likely	Very Likely
**The overall risk of injury in youth soccer **							
*Coaches (n = 10)*	0	0	1 (10.0)	1 (10.0)	6 (60.0)	2 (2.0)	0
**Expect to sustain an injury during the season**							
*Players (n = 200)*	9 (4.5)	32 (16.0)	41 (20.5)	29 (14.5)	69 (34.5)	16 (8.0)	4 (2.0)
**Believe injuries are preventable**							
*Coaches (n = 10)*	0	0	1 (10.0)	1 (10.0)	8 (80.0)	0	0
*Players (n = 200)*	2 (1.0)	9 (4.5)	18 (9.0)	23 (11.5)	60 (30.0)	63 (31.5)	25 (12.5)
**Believe FIFA 11+ will reduce injury risk**							
*Coaches (n = 10)*	0	0	1 (10.0)	0	2 (20.0)	1 (10.0)	6 (6.0)
*Players (n = 200)*	1 (0.5)	6 (3.0)	8 (4.0)	25 (12.5)	46 (23.0)	81 (40.5)	33 (16.5)

**Table 3:** Perceived self-efficacy for coaches and players (1 = not at all confident, 7 = extremely confident).

Question	Coaches (*n* = 10)	Players (*n* = 200)
How Confident are You	Mean (SD)	Mean (SD)
…that you understand the program well enough to use it with your team?	5.0 (1.5)	-
…in your ability to follow coach instructions about completing the program?	-	5.4 (0.7)
…that you have the ability to use the program with your team?	4.8 (1.4)	-
…in your ability to complete the exercises in the program?	-	4.4 (1.2)
…that you could continue to use the program for the whole season?	4.5 (1.6)	4.9 (1.1)
…that, if you did not perform the program at one session, you could start using it again next time?	4.9 (1.5)	4.7 (1.3)
…that you could use the program if there was limited space at the soccer venue?	4.5 (1.5)	-
…that you could use the program even if your players did not enjoy it?	4.7 (1.6)	-
…that you could perform the program with 100% effort at every session all season?	-	4.6 (1.0)
…that, if you did not give 100% effort in one session, you could give 100% next time?	-	4.7 (1.3)

**Table 4:** Pearson correlations between coach risk perceptions, outcome expectancies, task self-efficacy, and intention to use the FIFA 11+.

	Overall Injury Risk (RP1) ^1^	Ankle Injury Severity (RP2) ^1^	Knee Injury Severity (RP3) ^1^	Injuries Are Preventable (OE1) ^2^	FIFA 11+ Prevents Injuries (OE2) ^2^	I Understand the FIFA 11+ (TSE1) ^3^	I Can Use the FIFA 11+ (TSE2) ^3^	Intention to Use the FIFA 11+ (INT) ^4^
**RP1**	1.00	-	-	-	-	-	-	-
**RP2**	0.68 *	1.00	-	-	-	-	-	-
**RP3**	0.80 *	0.79 *	1.00	-	-	-	-	-
**OE1**	0.70 *	0.74 *	0.78 *	1.00	-	-	-	-
**OE2**	−0.34	0.06	−0.16	−0.18	1.00	-	-	-
**TSE1**	0.17	0.17	0.53	0.00	−0.25	1.00	-	-
**TSE2**	0.06	0.18	0.46	−0.07	−0.13	0.96 *	1.00	-
**INT**	0.09	0.13	0.55	0.13	−0.24	0.84 *	0.89 *	1.00

^1^ “RP” = risk perception; ^2^ “OE” = outcome expectancy; ^3^ “TSE” = task self-efficacy”; ^4^ “INT” = intention. * significant at *p* ≤ 0.05.

## Supplementary Materials

The following are available online at www.mdpi.com/1660-4601/13/7/657/s1: Coach questionnaire, Player questionnaire.

## Abbreviations

The following abbreviations are used in this manuscript:

FIFA	Fédération Internationale de Football
HAPA	Health Action Process Approach
CI	confidence interval
SD	linear dichroism
SRMR	standardized root mean square residual
RMSEA	root mean square of error of approximation
CFI	comparative fit index
TLI	Tucker Lewis index

## Appendix

**Table A1:** Facilitators supporting the use of the FIFA 11+ program.

Facilitator	Coaches (*n* = 10)	Players (*n* = 200)
	Frequency (%)	Frequency (%)
11+ resources (posters, videos, etc.)	10 (100)	
A shorter program		9 (4.5)
Assistant coaches to lead the program	1 (10)	
Players being healthy		8 (4.0)
Being taught the exercises by an expert		16 (8.0)
Better player fitness	1 (10)	4 (2.0)
Challenging exercises		4 (2.0)
Doing it alone (not needing partners for exercises)		4 (2.0)
Doing it as a team		47 (23.5)
Easy program (not too tiring or physically demanding)		32 (16.0)
Enough space to accommodate the exercises	2 (20)	21 (10.5)
Equipment (cones, floor mats for indoor training sessions)	4 (40)	11 (5.5)
Fun or enjoyable exercises		47 (23.5)
Having enough time to complete the program	1 (10)	63 (31.5)
If the program was a better warm up (or included more stretching components)		50 (25.0)
Knowing the benefits of the program		12 (6.0)
Making the program more soccer-specific (more ball games)		6 (3.0)
Making it a team routine every session		58 (29.0)
Music to go with the program		12 (6.0)
Personal motivation/effort		71 (35.5)
Proper nutrition/hydration		6 (3.0)
Reward for doing the program		5 (2.5)
Strong team leadership (someone to set up and organize the team, someone to lead the exercises)		58 (29.0)
Team buy-in (players supporting the use of the program, players taking it seriously)	1 (10)	56 (28.0)
Variety in the exercises		7 (3.5)
Weather/field conditions		14 (7.0)
Miscellaneous		4 (2.0)

**Table A2:** Barriers to the use of the FIFA 11+ program.

Barrier	Coaches (*n* = 10)	Players (*n* = 200)
	Frequency (%)	Frequency (%)
Coach is away and no one else is willing to lead the program	2 (20)	
No music to go with the program		1 (0.5)
Not being taught the program well		30 (15.0)
Not challenging enough		5 (2.5)
Not enough or improper equipment to set up the program		5 (2.5)
Not enough space to accommodate the exercises	1 (10)	26 (13.0)
Not enough time to complete the program	4 (40)	34 (17.0)
Not enough variety in the exercises		3 (1.5)
Not fun or enjoyable to perform		27 (13.5)
Not knowing the benefits of the program		6 (3.0)
Not soccer-specific enough		4 (2.0)
Other misc		7 (3.5)
Players are always late (so there is not enough time to complete the program)	1 (10)	
Players are away or do not show up for training	2 (20)	17 (8.5)
Players injured/sick (unable to participate fully)		21 (10.5)
Poor nutrition/hydration		9 (4.5)
Poor personal motivation/effort		58 (29.0)
Poor team buy-in (players not supporting the use of the program, players not taking it seriously)	2 (20)	104 (52.0)
Poor team leadership (no one willing to set up and organize the team, no one to lead the exercises)		25 (12.5)
Poor weather/field conditions	1 (10)	28 (14.0)
Program causes injury/pain		5 (2.5)
Program duplicates or conflicts with existing training		4 (2.0)
Program is a poor warm up		24 (12.0)
Program is not a team routine every session		43 (21.5)
Program is too difficult/tiring		71 (35.5)
Program is too long		23 (11.5)
Program is used as punishment in training		3 (1.5)
Program negatively impacts performance	1 (10)	
