# Supplementary material for: Predictors of FIFA 11+ Implementation Intention in Female Adolescent Soccer: An Application of the Health Action Process Approach (HAPA) Model
Source: Int J Environ Res Public Health. 2016 Jul 7;13(7):657. doi: 10.3390/ijerph13070657 (PMC4962198; doi:10.3390/ijerph13070657)
Supplement: Supplementary file 1 [file ijerph-13-00657-s001.pdf]

UNIVERSITY OF CALGARY

COACH QUESTIONNAIRE

Name: ____________________________

Club & Team Name: _______________________

Date (dd/mm/yy): _______________________

Sport Injury Prevention Research Centre
UNIVERSITY OF CALGARY

Coach ID (office use only)

Thank you for agreeing to complete this survey. It should take you approximately 10 minutes to complete. Please answer all questions thoughtfully and as accurately as possible. Please ask the research assistant who gave you this survey to answer any questions you may have.

SECTION A: Coaching history

<u>Instructions</u>: Please answer the following questions and provide brief details where appropriate.

1. Have you upgraded or received any **soccer coaching certificates** or qualifications during the past season? If so, please describe the level of qualification, and certifying organization.

   ☐ No
   ☐ Yes, please describe: ____

2. Do you have **medical or first aid certificate** or qualification? If so, please describe your level of qualification, and certifying organization.

   ☐ No
   ☐ Yes, please describe: ____

3. During the past soccer season, did you use a specific conditioning program with your team at training sessions to **improve the fitness** of your players?

   ☐ No, please describe why:

   ____

   ____

   ☐ Yes……..Please describe briefly:

   ____

   ____

4. During the past soccer season, did you use a specific conditioning program with your team at training sessions to **reduce the risk of injuries** among your players?

   ☐ No, please describe why:

   ____

   ____

   ☐ Yes……..Please describe briefly:

   ____

   ____

COACH QUESTIONNAIRE

UNIVERSITY OF CALGARY

Sport Injury Prevention Research Centre
UNIVERSITY OF CALGARY

Name: ______________________________

Club & Team Name: _________________________

Date (dd/mm/yy): _________________________

Coach ID (office use only)

SECTION B: Coach beliefs and attitudes

Instructions: Please take a few minutes to tell us what you think about injury risk in youth soccer, and about completing a **the FIFA 11+ warm-up program with your team at every game and training session during the next soccer season.** There are no right or wrong responses; we are merely interested in your personal opinions.

1. In your opinion, **overall injury risk** in youth soccer is:

**Low**	1	2	3	4	5	6	7	**HIgh**
	Extremely	Quite	Slightly	Neither	Slightly	Quite	Extremely	

2. In your opinion, how serious are the following types of soccer injury?

	Not at all serious			Moderately serious			Very serious
Ankle sprain	**1**	**2**	**3**	**4**	**5**	**6**	**7**
Knee ligament injury	**1**	**2**	**3**	**4**	**5**	**6**	**7**
Muscle strain	**1**	**2**	**3**	**4**	**5**	**6**	**7**
Broken bone	**1**	**2**	**3**	**4**	**5**	**6**	**7**
Cut or scrape	**1**	**2**	**3**	**4**	**5**	**6**	**7**
Bruise	**1**	**2**	**3**	**4**	**5**	**6**	**7**
Concussion	**1**	**2**	**3**	**4**	**5**	**6**	**7**

3. In general, how **preventable** do you think soccer injuries are (please circle one)?

**Not Preventable**	1	2	3	4	5	6	7	**Preventable**
	Extremely	Quite	Slightly	Neither	Slightly	Quite	Extremely	

4. In your opinion, what would happen to a soccer player's **overall risk of injury** if he or she participated in the FIFA 11+ warm-up program?

**Decrease**	1	2	3	4	5	6	7	**Increase**
	Extremely	Quite	Slightly	Neither	Slightly	Quite	Extremely	

UNIVERSITY OF CALGARY

COACH QUESTIONNAIRE

Name: ______________________________

Club & Team Name: ________________________

Date (dd/mm/yy): ________________________

Sport Injury Prevention Research Centre
UNIVERSITY OF CALGARY

Coach ID (office use only)

5. How confident are you that you **understand** the FIFA 11+ warm-up program well enough to use it with the team(s) you are coaching in the upcoming outdoor season?

Not Confident	1	2	3	4	5	6	7	Confident
	Extremely	Quite	Slightly	Neither	Slightly	Quite	Extremely	

6. How confident are you that you **have the ability** to use the FIFA 11+ warm-up program with the team(s) you are coaching in the upcoming outdoor season?

Not Confident	1	2	3	4	5	6	7	Confident
	Extremely	Quite	Slightly	Neither	Slightly	Quite	Extremely	

7. What **resources are available** to help you complete the FIFA 11+ warm-up program with your team at every game and training session during the next soccer season?
   i.____
   ii.____
   iii.____
   iv.____
   v.____

8. What **potential barriers** may prevent you from completing the FIFA 11+ warm-up program with your team at every game and training session during the next soccer season?
   i.____
   ii.____
   iii.____
   iv.____
   v.____

9. If there was limited space at your practice or game venue, how confident are you that your team could complete the FIFA 11+ warm-up program?

Not Confident	1	2	3	4	5	6	7	Confident
	Extremely	Quite	Slightly	Neither	Slightly	Quite	Extremely	

UNIVERSITY OF CALGARY

COACH QUESTIONNAIRE

Name: ______________________________

Club & Team Name: ________________________

Date (dd/mm/yy): ________________________

Sport Injury Prevention Research Centre
UNIVERSITY OF CALGARY

Coach ID (office use only)

10. If the players on your team did not enjoy performing the FIFA 11+ warm-up program, how confident are you that you could still have them complete it at every game and training session?

**Not Confident**	1	2	3	4	5	6	7	**Confident**
	Extremely	Quite	Slightly	Neither	Slightly	Quite	Extremely	

11. If you were to start the season by completing the FIFA 11+ warm-up program with your team at every game and training session, how confident are you that you could continue to do so for the entire season?

**Not Confident**	1	2	3	4	5	6	7	**Confident**
	Extremely	Quite	Slightly	Neither	Slightly	Quite	Extremely	

12. Imagine that your team regularly completes the FIFA 11+ warm-up program at games and training sessions. If you missed completing the program at a game or practice, how confident are you that you could start completing it again at the next session?

**Not Confident**	1	2	3	4	5	6	7	**Confident**
	Extremely	Quite	Slightly	Neither	Slightly	Quite	Extremely	

13. Do you intend to make injury prevention a priority for your team in the next soccer season?

**Not Likely**	1	2	3	4	5	6	7	**Likely**
	Extremely	Quite	Slightly	Neither	Slightly	Quite	Extremely	

14. Do you intend to complete the FIFA 11+ warm-up program with your team at every game and training session during the next soccer season?

**Not Likely**	1	2	3	4	5	6	7	**Likely**
	Extremely	Quite	Slightly	Neither	Slightly	Quite	Extremely	

UNIVERSITY OF CALGARY

COACH QUESTIONNAIRE

Name: ______________________________

Club & Team Name: _________________________

Date (dd/mm/yy): _________________________

Sport Injury Prevention Research Centre
UNIVERSITY OF CALGARY

Coach ID (office use only)

15. Do you already have concrete plans …

a. How to teach the FIFA 11+ warm-up program to the players on your team?

	1	2	3	4	5	6	7	
**Disagree**	Extremely	Quite	Slightly	Neither	Slightly	Quite	Extremely	**Agree**

b. When to have your team complete the FIFA 11+ warm-up program?

	1	2	3	4	5	6	7	
**Disagree**	Extremely	Quite	Slightly	Neither	Slightly	Quite	Extremely	**Agree**

16. Do you already have concrete plans …

a. How to deal with challenges in order to stick to your intentions of completing the FIFA 11+ warm-up program?

	1	2	3	4	5	6	7	
**Disagree**	Extremely	Quite	Slightly	Neither	Slightly	Quite	Extremely	**Agree**

b. How to encourage your team to perform the FIFA 11+ warm-up program to the best of their ability?

	1	2	3	4	5	6	7	
**Disagree**	Extremely	Quite	Slightly	Neither	Slightly	Quite	Extremely	**Agree**

COACH QUESTIONNAIRE

Name: ____________________________

Club & Team Name: ______________________

Date (dd/mm/yy): ______________________

Coach ID (office use only)

Thank you for agreeing to complete this survey. It should take you approximately 10-15 minutes to complete. Please answer all questions thoughtfully and as accurately as possible. Please ask the research assistant who gave you this survey to answer any questions you may have.

SECTION A: Players Participation and Injury History.

Instructions: Please answer the following questions and provide brief details where appropriate.

Previous Soccer Playing History

1. At which level of play did you play in **last soccer season**? *(indicate more than one if appropriate)*
   - ☐ Recreational or social league
   - ☐ Club → ☐ Tier I ☐ Tier 2 ☐ Tier3 ☐ Tier 4 ☐ Tier 5 ☐ Tier 6
   - ☐ Provincial team
   - ☐ National team
   - ☐ Other, please specify________________
2. Have you ever played on a soccer team that used a specific conditioning program at training sessions to **improve players' fitness**?
   - ☐ No
   - ☐ Yes……..Please describe briefly ____

     ____
3. Have you ever played on a soccer team that used a specific conditioning program at training sessions to **reduce players' risk of injuries**?
   - ☐ No
   - ☐ Yes……..Please describe briefly ____

     ____

COACH QUESTIONNAIRE

UNIVERSITY OF CALGARY

Sport Injury Prevention Research Centre
UNIVERSITY OF CALGARY

Name: ______________________________

Club & Team Name: ________________________

Date (dd/mm/yy): ________________________

Coach ID (office use only)

Previous Soccer Injury History

4. In the past 12 months, did you experience a lower extremity injury (e.g. foot, ankle, calf, shin, knee, groin, thigh or hip) from playing soccer which **resulted in you not being able to participate in a game or training session**?

☐ No

☐ Yes……..Please describe to the best of your ability the following details about the **MOST SEVERE** injury you had

Approximate date of injury (month & year):______________________________

Body part:______________________________

Type of injury:______________________________

How did it occur:______________________________

How long were you unable to participate in soccer practice or games?______________________________

5. In the past 12 months, did you experience a lower extremity injury (e.g. foot, ankle, calf, shin, knee, groin, thigh or hip) from playing soccer which **resulted in you not being able to attend school or work for at least one day**?

☐ No

☐ Yes……..Please describe to the best of your ability the following details about the **MOST SEVERE** injury you had

Approximate date of injury (month & year):______________________________

Bodypart:______________________________

Type of injury:______________________________

How did it occur:______________________________

How long were you unable to attend work or school?______________________________

SECTION B: Players Beliefs and Attitudes

Section B of this survey asks you questions about your attitudes and feelings towards **completing the FIFA 11+ warm-up program with your team at every game and training session during the next soccer season.**

The questions use a rating scale with 7 places. You need to circle the number that best describes what you think. For example, if you were asked to rate "The weather in Calgary" and you think it is "extremely good" then you would circle the *number 7*, like this:

COACH QUESTIONNAIRE

UNIVERSITY OF CALGARY

Sport Injury Prevention Research Centre
UNIVERSITY OF CALGARY

Name: ______________________________

Club & Team Name: _________________________

Date (dd/mm/yy): _________________________

Coach ID (office use only)

The weather in Calgary is

**Bad**	1	2	3	4	5	6	7 (circled)	**Good**
	Extremely	Quite	Slightly	Neither	Slightly	Quite	Extremely	

- ***Please make sure you answer all the items – don't leave any out.***
- ***Only circle one number for each question***
- ***Please do not circle in between the numbers***

1. In your opinion, how serious would it be if you experienced the following types of soccer injury?

	**Not at all serious**			**Moderately serious**			**Very serious**
Ankle sprain	**1**	**2**	**3**	**4**	**5**	**6**	**7**
Knee ligament injury	**1**	**2**	**3**	**4**	**5**	**6**	**7**
Muscle strain	**1**	**2**	**3**	**4**	**5**	**6**	**7**
Broken bone	**1**	**2**	**3**	**4**	**5**	**6**	**7**
Cut or scrape	**1**	**2**	**3**	**4**	**5**	**6**	**7**
Bruise	**1**	**2**	**3**	**4**	**5**	**6**	**7**
Concussion	**1**	**2**	**3**	**4**	**5**	**6**	**7**

2. I expect I will sustain an injury sometime during the next soccer season *(circle one)*

**Likely**	1	2	3	4	5	6	7	**Unlikely**
	Extremely	Quite	Slightly	Neither	Slightly	Quite	Extremely	

3. Many soccer injuries are preventable (*circle one*)

**False**	1	2	3	4	5	6	7	**True**
	Extremely	Quite	Slightly	Neither	Slightly	Quite	Extremely	

4. Completing the FIFA 11+ warm-up program with my team at every game and training session during this soccer season will reduce my risk of sustaining an injury *(circle one)*

**False**	1	2	3	4	5	6	7	**True**
	Extremely	Quite	Slightly	Neither	Slightly	Quite	Extremely	

COACH QUESTIONNAIRE

UNIVERSITY OF CALGARY

Sport Injury Prevention Research Centre
UNIVERSITY OF CALGARY

Name: ______________________________

Club & Team Name: ________________________

Date (dd/mm/yy): ________________________

Coach ID (office use only)

5. How confident are you that you are able to properly complete the exercises in the FIFA 11+ warm-up program? (*circle one*)

**Not Confident**	1	2	3	4	5	6	7	**Confident**
	Extremely	Quite	Slightly	Neither	Slightly	Quite	Extremely	

6. How confident are you that you are able to follow your coach's instructions about completing the FIFA 11+ warm-up program? (*circle one*)

**Not Confident**	1	2	3	4	5	6	7	**Confident**
	Extremely	Quite	Slightly	Neither	Slightly	Quite	Extremely	

7. If you were to start the season by completing the FIFA 11+ warm-up program with your team at every game and training session, how confident are you that you could continue to do so for the entire season? *(circle one)*

**Not Confident**	1	2	3	4	5	6	7	**Confident**
	Extremely	Quite	Slightly	Neither	Slightly	Quite	Extremely	

8. How confident are you that you could perform the exercises in the FIFA 11+ warm-up program with 100% effort at every game and training session? (*circle one*)

**Not Confident**	1	2	3	4	5	6	7	**Confident**
	Extremely	Quite	Slightly	Neither	Slightly	Quite	Extremely	

9. If you didn't perform the exercises with 100% effort at one game or training session, how confident are you that you could perform them with 100% effort the next time? (*circle one*)

**Not Confident**	1	2	3	4	5	6	7	**Confident**
	Extremely	Quite	Slightly	Neither	Slightly	Quite	Extremely	

COACH QUESTIONNAIRE

Name: ______________________________

Club & Team Name: ________________________

Date (dd/mm/yy): ________________________

Coach ID (office use only)

10. If your team started the season by completing the FIFA 11+ warm-up program at every game and training session, but then didn't do it at one game or practice, how confident are you that you would be able to start doing it again? *(circle one)*

**Not Confident**	1	2	3	4	5	6	7	**Confident**
	Extremely	Quite	Slightly	Neither	Slightly	Quite	Extremely	

11. I intend to complete the FIFA 11+ warm-up program at every game and training session during the next soccer season. *(circle one)*

**Disagree**	1	2	3	4	5	6	7	**Agree**
	Extremely		Quite	Slightly	Neither	Slightly	Quite	Extremely

12. If my team uses the FIFA 11+ warm-up plan during the next soccer season, I intend to complete the exercises with 100% effort. *(circle one)*

**Disagree**	1	2	3	4	5	6	7	**Agree**
	Extremely	Quite	Slightly	Neither	Slightly	Quite	Extremely	

13. Do you already have specific plans about how to complete the FIFA 11+ warm-up program before games and training sessions? *(circle one)*

**Disagree**	1	2	3	4	5	6	7	**Agree**
	Extremely	Quite	Slightly	Neither	Slightly	Quite	Extremely	

14. Do you already have specific plans about when to complete the FIFA 11+ warm-up program next season? *(circle one)*

**Disagree**	1	2	3	4	5	6	7	**Agree**
	Extremely	Quite	Slightly	Neither	Slightly	Quite	Extremely	

15. Do you already have specific plans about how to complete the FIFA 11+ warm-up program before games and training sessions, even if you don't like doing it? *(circle one)*

**Disagree**	1	2	3	4	5	6	7	**Agree**
	Extremely	Quite	Slightly	Neither	Slightly	Quite	Extremely	

UNIVERSITY OF CALGARY

COACH QUESTIONNAIRE

Name: ______________________________

Club & Team Name: ________________________

Date (dd/mm/yy): ________________________

Sport Injury Prevention Research Centre
UNIVERSITY OF CALGARY

Coach ID (office use only)

16. Do you already have specific plans about how to complete the FIFA 11+ warm-up program before games and training sessions, even if it is difficult to find space or time to do it? *(circle one)*

Disagree	1	2	3	4	5	6	7	Agree
	Extremely	Quite	Slightly	Neither	Slightly	Quite	Extremely	

17. What things would **make it easier** for you to complete the FIFA 11+ warm-up program with your team at every game and training session during the next soccer season?
    i.____
    ii.____
    iii.____
    iv.____
    v.____

18. What things would **make it harder** for you to complete the FIFA 11+ warm-up program with your team at every game and training session during the next soccer season?
    i.____
    ii.____
    iii.____
    iv.____
    v.____
